# Editorial: Next generation *in vitro* models to study chronic pulmonary diseases

**DOI:** 10.3389/fmed.2023.1338939

**Published:** 2023-12-05

**Authors:** Fama Thiam, Alen Faiz, Simon D. Pouwels, Emmanuel T. Osei

**Affiliations:** ^1^Department of Biology, University of British Columbia, Kelowna, BC, Canada; ^2^Faculty of Science, Respiratory Bioinformatics and Molecular Biology, University of Technology Sydney, Sydney, NSW, Australia; ^3^Department of Pulmonology, University Medical Center Groningen, University of Groningen, Groningen, Netherlands; ^4^Department of Pathology and Medical Biology, University Medical Center Groningen, University of Groningen, Groningen, Netherlands; ^5^University Medical Center Groningen, Groningen Research Institute for Asthma and Chronic Obstructive Pulmonary Disease, University of Groningen, Groningen, Netherlands; ^6^Centre for Heart Lung Innovation, St. Paul's Hospital, Vancouver, BC, Canada

**Keywords:** *in vitro* models, chronic pulmonary disease, air liquid interface (ALI), precision cut lung slice, CAD modeling, *ex vivo* models

Chronic pulmonary diseases, such as chronic obstructive pulmonary disease (COPD), tuberculosis (TB), and idiopathic pulmonary fibrosis (IPF), impact millions of people and are leading causes of death worldwide (1–3). Hence, a vast amount of research effort has been made to find early detection and curative therapies for these diseases. Researchers utilize several novel *in vitro* and *ex vivo* models to investigate the underlying mechanisms behind respiratory diseases. This is intended to help create targeted treatments that can aid in assessing and understanding novel disease mechanisms to subsequently improve the prognosis and quality of life of patients living with chronic pulmonary illnesses.

The use of bioinformatics and surgical models to improve diagnostics in respiratory diseases has the potential to impact clinical outcomes. In this Research Topic, studies from Oloko-Oba and Viriri and Gupta et al. assessed computer-aided diagnostic (CAD) systems related to TB diagnosis from the common and sensitive chest X-ray (CXR), which use deep learning techniques as well as a cellulose matrix absorptive probe for bronchial epithelial lining fluid (bELF) in the airways, respectively. From the systematic review of Oloko-Oba and Viriri, it was found that although most studies were developmental instead of being used in the clinic, the use of public and training datasets presented great potential for the use of CAD systems to improve TB diagnosis. In line with lung disease clinical diagnostics, Gupta et al. also discovered that the newly established bELF probes maintained their integrity with no residual fibers *in vivo* and obtained samples rich in proteins and with higher levels of inflammatory cytokines compared to the samples obtained from bronchial wash fluid. Ultimately, this high-precision probe is a novel technique for analyzing biomarkers in a consistent and accurate manner that will aid in early detection of lung disease (Gupta et al.).

In addition to diagnostics, air liquid interface (ALI) cultured cells have been used to assess different factors in disease severity, which is necessary to advance therapeutic treatments. In this Research Topic, Ito et al. used ALIs to show that age was a significant factor affecting respiratory syncytial virus (RSV) infection, with increased viral load and viral genome copies, lower viral clearance, higher inflammation, increased cell damage, mucin production, and cellular senescence in ALIs of older people (>65 years) compared to younger individuals (≤ 60 years). Additionally, Kasper et al. compared an ALI model to a submerged culture model to show that SARS-CoV-2 entry genes, such as angiotensin converting enzyme 2 (ACE2), transmembrane serine protease 2 (TMPRSS2), cathepsin L (CTSL), and tyrosine protein kinase receptor UFO (AXL) in human primary small airway epithelial cells (SAEC) or bronchial epithelial cells (HBEC), are affected more by culture conditions than individual donor conditions.

Lung organoids are another *in vitro* model that is used to assess cell mechanisms and their implications in pulmonary diseases [Wisman et al.; (4, 5)]. Here, Wisman et al. developed organoids of MRC-5 and unfractionated lung cell suspensions or isolated EpCAM^+^ distal lung tissue pulmonary epithelial cells from individuals with/without IPF. Organoids from IPF-derived cells were larger compared to isolated EpCAM^+^ cell-organoids, suggesting intrinsic progenitor dysfunction. Unfractionated cell suspensions from IPF-derived lungs also resulted in a higher number of organoids, suggesting a dysregulated communication between epithelial and stroma cells in IPF, which may lead to distal lung alveolar impairment (Wisman et al.).

Another important model is the precision cut lung slice (PCLS) model, where thin slices of lung tissue are cultured *in vitro* for studies into disease mechanisms (6, 7). In this Research Topic, Cervantes et al. established PCLS from donors without a history of disease and exposed them to particulate matter from Afghanistan (PMa) (8) or particulate matter from California as a control (PMc) to investigate the mechanisms related to unique military deployment airway symptoms. Interestingly, PCLS was used to show that PMa increased airway hyperresponsiveness (AHR), but PMc had no effect (Cervantes et al.). Additionally, PMa co-stimulated with IL-13 resulted in significantly amplified AHR compared to PMc co-stimulations (Cervantes et al.).

Another important mechanism underlying respiratory disease pathogenesis is the disruption of the epithelial barrier integrity (9). Hsieh et al. used the Electric Cell-substrate Impedance Sensing (ECIS) system to assess the effect of different ECM substrates on the barrier integrity and attachment of basal airway epithelial cells. It was shown that airway epithelial cells attached faster on fibronectin, collagen I, and collagen III than on collagen IV and laminin. Further, fibronectin and collagen I enabled the fastest epithelial barrier formation compared to the other ECM proteins. This study demonstrated a potential protective role of these ECM proteins in pathological lung conditions (Hsieh et al.).

Respiratory cancers are a prominent source of cancer incidence and mortality worldwide, therefore investigations into their mechanisms of drug resistance are essential to identify novel treatment targets (10, 11). Tuffour et al. used CRISPER-Cas-9 gene editing to knockout the *CASD1* and *SIAE* genes, which are an important part of the breast cancer resistance protein (BCRP), a main ATP-binding cassette (ABC) transporter protein involved in multidrug resistant (MDR) pathways. Here, using CRISPR-gene editing and drug sensitivity analysis, it was shown that deacetylated Sias are utilized by cancer cells to overexpress BCRP as a pathway of MDR, which can be used to further advance the effectiveness of chemotherapies.

*Ex vivo* models have also been utilized to study the biological methods of disease and potentially identify mechanisms for therapeutics due to their ability to mimic the *in vivo* physiology. Ievlev et al. created a ferret tracheal model for injury and cell engraftment using tracheal explants and found a semblance to surface airway epithelium (SAE) and submucosal glands (SMGs). Consistent results in line with published data on *in vivo* injury systems were found after injury experiments. A 3D-printed culture chamber that allows imaging of ferret tissue explants was set up, which aided ferret cell ALI establishment.

In this Research Topic, various studies utilized a breadth of tools including CAD modeling, pulmonary sampling devices, ALIs, PCLS, ECIS, CRISPR, and *ex vivo* systems to study different mechanisms of pulmonary diseases as well as diagnostics and to perform drug studies. These prove the utility and adaptability of these systems for future studies and the advancement of the pulmonary field.

## Author contributions

FT: Writing – original draft, Writing – review & editing. AF: Conceptualization, Writing – review & editing. SP: Conceptualization, Writing – review & editing. EO: Conceptualization, Writing – original draft, Writing – review & editing.
